# Too hot to move? Objectively assessed seasonal changes in Australian children’s physical activity

**DOI:** 10.1186/s12966-015-0245-x

**Published:** 2015-06-19

**Authors:** Nicola D. Ridgers, Jo Salmon, Anna Timperio

**Affiliations:** Centre for Physical Activity and Nutrition Research, Deakin University, 221 Burwood Highway, Burwood, VIC 3125 Australia

**Keywords:** Seasons, Climate, Accelerometry, Youth

## Abstract

**Background:**

Seasonal variations may influence children’s physical activity patterns. The aim of this study was to examine how children’s objectively-measured physical activity differed across seasons, and whether different seasonal patterns were observed for boys and girls.

**Methods:**

Three hundred and twenty-six children aged 8–11 years from nine primary schools in Melbourne, Australia, participated in the study. Physical activity was measured every 15-s using hip-mounted GT3X+ ActiGraph accelerometers for seven consecutive days in the Winter (*n* = 249), Spring (*n* = 221), Summer (*n* = 174) and Autumn (*n* = 152) school terms. Time spent in moderate (MPA), vigorous (VPA) and moderate- to vigorous-intensity physical activity (MVPA) at each time point was derived using age-specific cut-points. Meteorological data (maximum temperature, precipitation, daylight hours) were obtained daily during each season. Longitudinal data were analysed using multilevel analyses, adjusted for age, sex, accelerometer wear time, number of valid days, and meteorological variables.

**Results:**

Compared to Winter, children engaged in significantly less MPA (−5.0 min) and MVPA (−7.8 min) in Summer. Girls engaged in less MVPA in Spring (−18 min) and Summer (−9.2 min) and more MVPA in Autumn (9.9 min) compared to Winter. Significant changes in MPA and VPA bout frequency and duration were also observed. Significant decreases in VPA bout frequency (3.4 bouts) and duration (2.6 min) were observed for girls in Spring compared to Winter. No significant seasonal changes were observed for boys for all intensities and physical activity accumulation.

**Conclusions:**

Physical activity decreased in Summer compared to Winter, contrasting previous research that typically reports that children are most active in summer. Greater fluctuations were observed for girls’ activity levels. In addition, girls’ activity duration and bouts appeared to be more susceptible to seasonal changes compared to boys. The results suggest that strategies to promote physical activity may be needed in Australia during the hot summer months, particularly for girls.

## Introduction

A range of beneficial effects of regular physical activity on children’s mental, social and physical health have been documented [1]. However, population estimates indicate that a large proportion of youth fail to engage in the recommended 60 min of moderate- to vigorous-intensity physical activity (MVPA) every day [2]. In Australia, only 22 % of boys and 20 % of girls aged 9–11 years are sufficiently active, and this proportion decreases with increasing age [3]. Understanding key influences is important for the development of effective strategies to promote children’s physical activity.

An area that has been infrequently studied is how season impacts on children’s naturally-occurring physical activity patterns. Weather patterns and daylight hours vary by season and this has been found to have a direct effect on physical activity [4]. Previous research in the United States and the United Kingdom, for example, has documented fluctuations in fitness levels and physical activity across seasons, with peaks typically occurring in summer months compared to winter months [5–11]. These fluctuations, which have been largely consistent in studies conducted around the world [7, 8], may have detrimental effects on people’s health. For example, obesity prevalence has been found to be higher in winter than summer [12, 13]. It has been suggested that higher levels of physical activity during the summer months may be due to increased opportunity with longer daylight hours and favourable weather conditions, which are conducive to being active outdoors [6, 9, 14].

However, the existing evidence base has a number of important limitations, including infrequent use of repeated measures designs to assess seasonal variation and a lack of consideration of each of the four seasons [8]. Studies typically examine cross-sectional differences between Summer and Winter, which does not provide a complete picture of children’s physical activity and limits the ability to identify key times of the year in which to intervene [14]. Furthermore, such designs do not permit an examination as to whether different seasonal patterns are observed for different population groups (e.g. boys and girls). A further limitation of the existing evidence base is that it relies predominantly on subjective measurement tools [7, 10, 11], which have limited utility in children [15]. Objective monitors, such as accelerometers, more accurately assess children’s physical activity levels and provide information concerning how activity is accumulated (e.g. intensity, activity bouts) [15]. However, changes in MVPA are typically reported [8], which may mask changes in moderate- (MPA) and vigorous-intensity (VPA) physical activity and the identification of which intensities may need to be targeted at specific times of the year to increase children’s activity levels. Lastly, few studies have controlled for meteorological information, even though daily fluctuations in these variables may affect physical activity within seasons [16, 17].

The aim of this study was to examine how children’s objectively-measured physical activity differed across seasons. A secondary aim was to determine whether different seasonal patterns were observed for boys and girls.

## Methods

### Sample

Primary schools enrolments greater than 200 pupils and located within a 40 km radius of the Melbourne Central Business District, Australia, were stratified into tertiles of socioeconomic status (SES) based on postcode using the Socio-Economic Index for Areas [18]. Schools were randomly selected from each SES strata and invited to participate in this study. Nine schools (1 low SES, 3 medium SES, and 5 high SES) agreed to participate and the School Principal provided informed consent. Ethical approval was provided by the Deakin University Human Ethics Advisory Group (Health), the Department of Education and Early Childhood Development, and the Catholic Education Office (Melbourne).

All children (*n* = 1270) in Years 4 and 5 (aged 8–11 years) were invited to participate in the Patterns of Habitual Activity across Seasons (PHASE) Study, which used a repeated measures design to examine seasonal changes in physical activity. Informed written parental consent was provided for 326 children (162 boys, 164 girls) to participate in at least one component of the study at baseline. As it is an ethics requirement in Australia for active informed consent to be provided, no information was obtained concerning non-responders. Data were collected during four different school terms across one calendar year that corresponded with each of the seasons in south-east Australia: Winter (August-September 2012; Term 3); Spring (October-November 2012; Term 4); Summer (February-March 2013; Term 1); and Autumn (May-June 2013; Term 2). In Melbourne (37.81 °S, 144.97 °E), the average maximum temperature and rainfall in the warmer months (October-March) range from 19.7 to 25.9 °C and 47.1 to 66.1 mm/month, respectively. The mean number of days of rain ranges from 5.1 to 10.2 days/month. In the cooler months (April-September), the average maximum temperature and rainfall range from 13.5 to 20.3 °C and 47.7 to 58.2 mm/month, respectively. The mean number of days of rain ranges from 7.9 to 10.5 days/month [19].

### Measures

#### Physical activity

Children’s physical activity levels were objectively measured for 7 consecutive days at each time point using a hip-mounted ActiGraph model GT3X+ (Pensacola, FL), which samples acceleration data using a 12 bit analogue converter at a user-specified rate (30–100 Hz) and is stored in the non-volatile flash memory [20]. Raw tri-axial acceleration data were sampled at 30 Hz in this study. Children were fitted with the monitors by trained researchers during class time, and instructed to wear the monitor during all waking hours except during water-based activities (e.g. swimming, bathing). Children were also provided with verbal and written information concerning the correct wear and care of the monitor at this time. The ActiGraph has acceptable reliability and validity in paediatric populations [21].

ActiGraph data were downloaded using ActiLife software (ActiGraph, Pensacola, FL) and processed using a customized Excel macro. Sustained periods of 20 min of consecutive zeros were used to define non-wear time [22], and the total duration of these periods represented the total duration of non-wear time. Time spent in moderate- (MPA; 4–5.99 METs) and vigorous- (VPA; ≥6 METs) intensity physical activity was determined using age-specific cut-points [23]. These cut-points have been found to exhibit excellent classification accuracy and are commonly used [24], thus enabling comparisons with previous research. A threshold of 4 METs was chosen to represent MPA as brisk walking has been associated with an energy cost of 4 METs in calibration studies with children [24]. Time spent in MPA and VPA were summed to obtain MVPA, which has also demonstrated excellent classification accuracy [24]. Bouts of ≥1 min of MPA and VPA were also calculated. The frequency of and duration of time accumulated in MPA and VPA bouts lasting ≥1 min were determined. No interruption periods to these bouts were permitted. This minimum bout threshold has previously been used to identify sporadic bouts of physical activity, which accounted for approximately two-thirds of children’s daily activity [25]. A valid day was defined as ≥8 h on weekdays and ≥7 h on weekend days [26]. The lower weekend wear time requirement is due to children typically waking later on weekends. To be included in the analyses, children were required to have worn the ActiGraph on at least three days, which has been shown to achieve a reliability coefficient of 0.7 for both valid day definitions [26].

Three hundred and eleven children (156 boys, 155 girls) wore accelerometers in Winter (August and September 2012), with complete data (based on the inclusion criteria) collected from 249 children (122 boys, 127 girls; 80 % of those who wore a monitor). Complete accelerometer data were obtained from 221 children in Spring (77 % of those who wore a monitor), 174 in Summer (64 %) and 152 in Autumn (58 %). Ninety-six children had complete data across the four seasons, whilst 74, 70, and 50 children provided data from three, two and one seasons for use in the analyses, respectively.

### Meteorological information

Meteorological data were obtained on each day of data collection at each time point from the publically accessible Australian Government’s Bureau of Meteorology website (www.bom.gov.au). This information was obtained from the weather station located nearest to each participating school and matched to accelerometer wear days. Data concerning the maximum recorded daily temperature (in degrees Celsius), whether it rained (yes/no) and, if so, total daily rainfall (in millimetres), and minutes of daylight per day were obtained. These data were averaged for each time point for use in the subsequent analyses.

### Statistical analyses

Descriptive statistics (mean ± SD) were initially calculated for measured variables. Multilevel models were used to examine differences in children’s physical activity levels across seasons. Multilevel models are the most appropriate technique for analysing nested data that are not independent of each other (e.g. terms nested within children within schools) and violate the assumption of independent associations [27]. As multilevel models do not require complete data sets for analyses, are robust against missing data points and can estimate effects over time using incomplete data sets [28], all collected data that met the inclusion criteria at each time point were used in the analyses (796 data points).

A three-level model was used in the analyses, where the levels were season of measurement (Level 1), child (Level 2) and school (Level 3). To estimate changes in children’s physical activity levels across seasons, three dummy predictor variables were generated. These were for physical activity levels during the Spring, Summer and Autumn terms compared to the Winter Term. Two models were constructed for each outcome variable, which were time spent in MPA, VPA, MVPA, bouts of MPA and VPA (min), and the frequency of MPA and VPA bouts. The first model adjusted for sex, age, number of valid days, and accelerometer wear time. The second model additionally adjusted for average maximum temperature, number of rain days, average rainfall, and average daylight minutes to examine whether these variables confounded any observed changes [16]. Examining seasonal patterns for boys and girls separately was identified a priori. Regression coefficients in the models were assessed for significance using the Wald statistic. Statistical significance was set at *p* < 0.05. Multilevel analyses were conducted using Stata SE version 12 (StataCorp LP, College Station, Texas).

## Results

Demographic information and the physical activity levels of the children during Winter (baseline measure; mean, SD) are presented in Table 1. Boys engaged in significantly more MPA, VPA and MVPA, and had a higher frequency of MPA and VPA bouts and accumulated more time in these bouts than girls (all *p* < 0.01). Boys had a significantly lower accelerometer wear time than girls (*p* < 0.05). Descriptive information concerning the meteorological data collected during each season are presented in Table 2.

**Table 1:** Descriptive baseline data collected from children during August-September 2012 (Winter; mean (SD))

	Whole sample	Girls	Boys
Age (years)	10.0 (0.7)	10.1 (0.7)	10.0 (0.7)
Daily MPA (min)	50.6 (13.4)	46.0 (11.9)**	55.4 (13.1)**
Daily VPA (min)	25.1 (13.3)	20.4 (10.1)**	30.1 (14.5)**
Daily MVPA (min)	75.8 (24.5)	66.5 (21)**	85.5 (24.5)**
Daily wear time (min)	808.9 (156.8)	829.4 (170.6)*	787.7 (138.1)*
MPA bouts per day (frequency)	6.6 (2.7)	5.6 (2.1)**	7.7 (2.8)**
Average time in MPA bouts (min)	8.9 (4.3)	7.4 (3.3)**	10.4 (4.6)**
VPA bouts per day (frequency)	4.8 (4.2)	3.5 (2.8)**	6.1 (4.9)**
Average time in VPA bouts (min)	7.3 (7.5)	5.1 (4.5)**	9.6 (9.1)**

Sex differences: *p < 0.05; **p < 0.01

**Table 2:** Meteorological data from each season of measurement (mean (SD) and range)

	August-September 2012 (Winter Term)		October-November 2012 (Spring Term)		February-March 2013 (Summer Term)		May-June 2013 (Autumn Term)	
	Mean (SD)	Range	Mean (SD)	Range	Mean (SD)	Range	Mean (SD)	Range
Average maximum daily temperature (°C)	14.8 (1.3)	13.5–17.4	23.0 (2.1)	20.4–26.2	28.3 (4.5)	22.3–32.8	17.6 (2.4)	15.1–21.4
Average daily rainfall (mm)	1.8 (1.1)	0.2–3.8	1.3 (1.4)	0.1–4.0	2.4 (2.4)	0–6.7	2.1 (1.8)	0.1–7.6
Average number of rainy days/week	5 (1.8)	3–7	2.5 (1.1)	1–4	2.6 (1.8)	0–5	3.7 (1.1)	2–6
Average daylight minutes	650.8 (21.4)	620.1–683.4	852.2 (17.9)	823.4–874.9	772.3 (32.6)	734–811.4	603.2 (21.5)	576–642.8

The results reporting seasonal changes in daily physical activity are presented in Table 3. The analyses show that children spent significantly less time in MPA and MVPA during summer compared to Winter (*p* < 0.05) after adjusting for potential meteorological confounders (Model 2). On average, children engaged in 5.0 min and 7.8 min less MPA and MVPA per day, respectively, in the Summer compared to the Winter. In the stratified analyses (Model 2), girls engaged in significantly more MPA and MVPA in Autumn compared to Winter. Significant decreases in VPA and MVPA were also found in the Spring and Summer terms, with the largest decrease in MVPA occurring in Spring (18 min) compared to Winter. No significant changes in boys’ physical activity levels were observed between seasons after additionally adjusting for meteorological variables.

**Table 3:** Changes in average daily physical activity (min) between the Winter school term (2012) and subsequent school terms (95 % CI)

	Model 1^a^			Model 2^b^		
	MPA	VPA	MVPA	MPA	VPA	MVPA
Whole sample						
Winter	Ref	Ref	Ref	Ref	Ref	Ref
Spring	2.4 (0.8 to 4.1)*	–2.4 (−3.6 to −1.2)**	−0.1 (−2.6 to 2.4)	−6.2 (−14.6 to 2.2)	−5.0 (−11.1 to 1.1)	*−*11.4 (−25.0 to 1.3)~
Summer	−4.4 (−6.3 to −2.4)**	−6.2 (−7.6 to −4.8)**	−10.8 (−13.7 to −7.8)**	−5.0 (−9.5 to −0.6)*	−2.7 (−5.9 to 0.4)~	−7.8 (−14.6 to −0.9)*
Autumn	−0.8 (−2.9 to 1.4)	−1.2 (−2.8 to 0.3)	−2.2 (−5.4 to 1.1)	3.8 (−0.6 to 8.2)~	2.6 (−0.7 to 5.8)	6.6 (−0.2 to 13.5)~
Boys						
Winter	Ref	Ref	Ref	Ref	Ref	Ref
Spring	4.2 (1.7 to 6.8)**	−1.6 (−3.5 to 0.4)	2.7 (−1.3 to 6.6)	−1.4 (−14.6 to 11.7)	−1.1 (−11.7 to 9.4)	−2.5 (−25.0 to 17.2)
Summer	−3.3 (−6.3 to −0.4)*	−6.2 (−8.5 to −3.9)**	−9.7 (−14.3 to −5.1)**	−5.1 (−12.3 to 2.1)	−1.8 (−7.2 to 3.8)	−7.0 (−17.9 to 3.9)
Autumn	−1.2 (−4.6 to 2.1)	−1.0 (−3.6 to 1.6)	−2.3 (−7.6 to 2.8)	0.8 (−6.6 to 7.1)	1.2 (−4.2 to 6.7)	2.1 (−8.9 to 13.2)
Girls						
Winter	Ref	Ref	Ref	Ref	Ref	Ref
Spring	0.7 (−1.5 to 2.8)	−2.9 (−4.4 to −1.5)**	−2.3 (−5.6 to 1.0)	−8.8 (−18.9 to 1.4)~	−8.9 (−15.7 to −1.6)*	−18.0 (−33.8 to −2.3)*
Summer	−5.4 (−7.8 to −2.9)**	−6.1 (−7.8 to −4.4)**	−11.4 (−15.2 to −7.7)**	−5.0 (−10.4 to 0.4)~	−4.2 (−7.9 to −0.5)*	−9.2 (−17.5 to −0.9)*
Autumn	−0.6 (−3.2 to 2.1)	−1.4 (−3.3 to 0.4)	−2.1 (−6.2 to 2.0)	5.8 (0.4 to 11.2)*	3.9 (−0.1 to 7.4)~	9.9 (1.5 to 18.2)*

*MPA* Moderate physical activity; *VPA* Vigorous physical activity; *MVPA* Moderate- to vigorous-intensity physical activity

~*p* <0.1; *p < 0.05; **p < 0.01

^a^Three level multilevel model (term, children, schools) adjusted for age, number of valid days, accelerometer wear time (whole sample additionally adjusted for sex)

^b^Three level multilevel model (term, children, schools) adjusted for age, number of valid days, accelerometer wear time, average maximum temperature, average daily rainfall, number of days of rain, and average daylight minutes during data collection (whole sample additionally adjusted for sex)

Table 4 reports changes in the frequency of MPA and VPA bouts and the total time accumulated in these bouts across seasons. These analyses revealed significant increases in MPA and VPA bout frequency and duration in Autumn compared to Winter for the whole sample and for girls (all *p* < 0.05). For example, children engaged in 2.5 more MPA bouts and spent 1.3 min longer in these bouts in Autumn compared to Winter. Significant decreases in VPA bout frequency (3.4 bouts) and duration (2.6 min) were also observed for girls in Spring compared to Winter. No significant changes in the accumulation of physical activity were observed for boys between seasons.

**Table 4:** Changes in average physical activity bout lengths and frequency between the Winter school term (2012) and subsequent school terms (95 % CI)

	Model 1^a^				Model 2^b^			
	MPA bouts (min)	MPA bouts (#)	VPA bouts (min)	VPA bouts (#)	MPA bouts (min)	MPA bouts (#)	VPA bouts (min)	VPA bouts (#)
Whole sample								
Winter	Ref	Ref	Ref	Ref	Ref	Ref	Ref	Ref
Spring	1.4 (0.6 to 2.2)**	0.8 (0.4 to 1.3)**	−1.1 (−1.8 to −0.5)**	−0.7 (−1.1 to −0.4)**	−1.5 (−5.3 to 2.4)	−1.0 (−3.2 to 1.1)	−2.8 (−6.2 to 0.6)	−1.8 (−3.7 to 0.2)~
Summer	0.6 (−0.3 to 1.5)	0.2 (−0.3 to 0.7)	−2.0 (−2.7 to −1.2)**	−1.3 (−1.7 to −0.9)**	−0.4 (−2.5 to 1.9)	−0.7 (−1.8 to 0.5)	−0.5 (−2.2 to 1.3)	−0.4 (−1.4 to 0.6)
Autumn	1.4 (0.4 to 2.4)*	0.6 (0.1 to 1.1)*	−0.3 (−1.2 to 0.6)	−0.1 (−0.6 to 0.4)	2.5 (0.4 to 4.5)**	1.3 (0.2 to 2.4)*	1.8 (0.1 to 3.6)*	1.2 (0.2 to 2.2)*
Boys								
Winter	Ref	Ref	Ref	Ref	Ref	Ref	Ref	Ref
Spring	1.2 (−0.1 to 2.5)~	0.8 (0.1 to 1.6)*	−1.2 (−2.4 to −0.1)*	−0.7 (−1.3 to −0.1)*	0.8 (−5.5 to 7.0)	0.7 (−3.0 to 4.4)	−2.6 (−9.0 to 3.7)	−0.9 (−4.4 to 2.6)
Summer	0.3 (−1.2 to 1.7)	0.1 (−0.8 to 0.9)	−2.5 (−3.9 to −1.1)**	−1.5 (−2.3 to −0.8)**	−0.6 (−4.1 to 2.9)	−0.3 (−2.4 to 1.7)	−1.3(−4.7 to 2.0)	−0.4 (−2.2 to 1.4)
Autumn	0.2 (−1.4 to 1.8)	0.2 (−0.8 to 1.1)	−0.2 (−1.8 to 1.4)	0.1 (−0.9 to 0.9)	−0.5 (−3.9 to 2.8)	−0.1 (−2.0 to 1.8)	1.5 (−1.8 to 4.8)	0.9 (−0.9 to 2.8)
Girls								
Winter	Ref	Ref	Ref	Ref	Ref	Ref	Ref	Ref
Spring	1.5 (0.6 to 2.5)**	0.8 (0.3 to 1.3)**	−0.9 (−1.6 to −0.2)*	−0.7 (−1.1 to −2.7)**	−2.8 (−7.4 to 1.6)	−2.1 (−4.6 to 0.3)~	−3.4 (−6.7 to −0.1)*	−2.6 (−4.6 to −0.6)*
Summer	0.8 (−0.3 to 1.9)	0.2 (−0.4 to 0.8)	−1.5 (−2.3 to −0.7)**	−1.1 (−1.6 to −0.6)**	−0.3 (−2.8 to 2.2)	−0.9 (−2.2 to 0.4)	−0.4 (−2.2 to 1.3)	−0.7 (−1.8 to 0.3)
Autumn	2.3 (1.1 to 3.5)**	0.9 (0.2 to 1.5)**	−0.3 (−1.2 to 0.5)	−0.2 (−0.8 to 0.3)	5.0 (2.6 to 7.3)**	2.4 (1.1 to 3.6)**	2.2 (0.5 to 4.0)*	1.4 (0.4 to 2.5)*

*MPA* Moderate physical activity; *VPA* Vigorous physical activity

~*p* <0.1; *p < 0.05; **p < 0.01

^a^Three level multilevel model (term, children, schools) adjusted for age, number of valid days, accelerometer wear time (whole sample additionally adjusted for sex)

^b^Three level multilevel model (term, children, schools) adjusted for age, number of valid days, accelerometer wear time, average maximum temperature, average daily rainfall, number of days of rain, and average daylight minutes during data collection (whole sample additionally adjusted for sex)

## Discussion

This study examined changes in children’s objectively measured physical activity duration and bouts across different seasons. The results showed that children were significantly less active in Summer compared to Winter after adjustment for meteorological variables in the analyses. Increases in the frequency and duration of MVPA bouts were also observed in Autumn compared to Winter.

The lower participation in physical activity levels observed in Summer compared to Winter across the whole sample contrasts with the published literature, which has typically observed higher activity levels in Summer compared to the rest of the year [7–9]. Only one study, to the best of our knowledge, has reported results consistent with the present study’s findings. Baranowski and colleagues [29] found that in an environment that experiences high Summer temperatures, similar to Australia, pre-schoolers outdoor play activity levels were lower in the Summer months compared to Autumn and Winter. In contrast, indoor play activity levels were less variable throughout the year [29]. It has been suggested that longer days and warmer temperatures facilitate physical activity behaviours in Summer, whilst adverse conditions such as higher rainfall and lower temperatures in Winter may be a barrier to physical activity [9]. It is possible in this study, however, that the higher temperatures experienced by the children in the Summer term may have acted as a potential barrier to physical activity. The higher activity levels observed in Winter may also be explained, in part, by the structured sports and activities in which children participate. A recent national survey in Australia reported that the top 10 popular organised sports for children included outdoor soccer (boys and girls), Australian Rules Football (boys) and netball (girls) [30], which are typically played in the Autumn and Winter months. Overall, the current study findings suggest that that strategies may be needed to promote children’s physical activity levels during the warmer Summer months.

Differences in daily physical activity between seasons were observed for girls but not boys. Previous research has demonstrated seasonal differences in girls’ physical activity levels, though our finding that girls were most active in Autumn and least active in Summer is not consistent with the literature [6, 14, 31, 32]. Similar seasonal patterns of change were observed for boys, though the differences were not significant, which supports the findings of Gracia-Marco and colleagues [32] who observed no differences in European adolescent boys’ activity levels between Spring and Winter. They hypothesised that greater preferences for outdoor activities and higher access to outdoor areas may be attributable to boys’ activity levels in the different seasons compared to girls [32]. Whilst time spent outdoors has been found to be positively associated with physical activity [33], and boys spend more time outdoors after school in both warmer and cooler months compared to girls [34], little research to date has examined whether seasonal changes occur throughout the day or during specific time periods such as after school. Further research is needed to identify modifiable and unmodifiable factors that may explain differences in physical activity across seasons, particularly for girls, and to identify whether seasonal changes occur during specific time periods of the day. Such information will inform the development of interventions to reduce seasonal fluctuations in physical activity.

Changes in the frequency and bouts of physical activity were also observed between seasons. In the whole sample, the duration and frequency of MPA bouts were higher in Autumn compared to Winter, though the stratified analyses suggest that this result was largely explained by changes among girls. Only one study, to the best of our knowledge, has examined changes in the pattern of physical activity accumulation between different seasons. Rowlands and colleagues [35] reported longer and more frequent bouts of physical activity in the Summer compared to the Winter for English boys and girls, which was not supported by the current study. It is possible the cooler months may be more favourable for girls to engage in more sustained periods of physical activity more frequently than the warmer months. Interestingly, the cooler months in this study were similar to the warmer months in Rowlands and colleagues [35] study in terms of average temperature. While the present study adjusted for meteorological differences, future research comparing physical activity levels between countries based on weather rather than seasons may be informative. Overall, the results suggest that both the frequency and duration of physical activity bouts are susceptible to change between seasons and could be manipulated by interventions that aim to increase physical activity levels during the times of the year in which children are less active.

The strengths of this study include the objective measurement of children’s physical activity levels across four seasons. There are, however, several limitations. Firstly, for feasibility reasons, the assessment of physical activity was conducted in the middle of the school terms. Whilst the school terms closely align with their respective seasons, the timing of school holidays meant that in some cases data were collected at the end of the season rather than the middle of the season, which may be more representative of the seasons being assessed. Secondly, accelerometers are limited in their ability to assess water-based physical activity. It is possible that physical activity levels during Summer were underestimated, though it should be noted that swimming is one of the most popular organised sports in Australia all year around [30]. Examining physical activity levels using waterproof wearable monitors may overcome this limitation in future studies. Thirdly, the inclusion criteria of 3 days did not require a weekend day in order to maximise the sample size. Although physical activity differs on weekday and weekend days [36], when analyses were repeated to require a weekend day, the results were similar (data not shown). Fourth, there were missing data in the study, particularly in Terms 1 and 2, which was largely attributable to non-compliance with the protocol. However, a strength of the analytical approach is that multilevel analyses are robust to missing data. Lastly, only one low SES school participated in the study; therefore the findings may reflect seasonal differences in medium and high SES children. Future research should examine whether similar seasonal differences are observed in low SES populations.

## Conclusion

The findings from this study suggest that children’s physical activity duration was lower in the Summer compared to Winter. In addition, girls’ activity duration and bouts appeared to be more susceptible to seasonal changes compared to boys. These results contrast previous research, often conducted in the Northern Hemisphere, what has suggested that children are more active in warmer months [6–8]. However, the direction of the findings are consistent with a previous study conducted in the US that was also conducted in a climate that experiences hot Summer temperatures [29]. The results suggest that strategies to promote physical activity may be needed in Australia during the hotter Spring and Summer months, particularly for girls, to maintain activity levels all year around. Further research is needed to establish the factors that may influence these seasonal differences in physical activity and the periods of the day where such changes occur to inform future intervention efforts.
